# Bracts, Buds, and Biases: Uncovering Gaps in Trichome Density Quantification and Cannabinoid Concentration in *Cannabis sativa* L.

**DOI:** 10.3390/plants14142220

**Published:** 2025-07-18

**Authors:** Thaís Alberti, Fardad Didaran, Shiksha Sharma, Rodrigo De Sarandy Raposo, Andre A. Diatta, Marcelo Maraschin, Jose F. Da Cunha Leme Filho

**Affiliations:** 1 School of Forestry and Horticulture, Southern Illinois University, Carbondale, IL 62901, USA; thais.barbosaalberti@siu.edu (T.A.); shiksha.sharma@siu.edu (S.S.); rodrigo.desarandyraposo@siu.edu (R.D.S.R.); 2 School of Biological Sciences, Southern Illinois University, Carbondale, IL 62901, USA; 3 Laboratory of Plant Morphogenesis and Biochemistry, Department of Plant Science, Federal University of Santa Catarina, Florianópolis 88034-000, SC, Brazil; m2@cca.ufsc.br; 4 Laboratory of Natural and Synthetic Products, Biotechnology Institute, University of Caxias do Sul, Caxias do Sul 95070-560, RS, Brazil; 5 Biomass Production Laboratory, Department of Bioresource Engineering, McGill University, Ste-Anne-de-Bellevue, QC H9X 3V9, Canada; f.didaran@ut.ac.ir; 6 Department of Agronomy, Gaston Berger University, Saint-Louis 32000, Senegal; andre-amakobo.diatta@ugb.edu.sn

**Keywords:** cannabinoids, terpenes, calyxes, anatomic analysis, plant structures, protocols

## Abstract

Trichomes in cannabis (*Cannabis sativa* L.) are specialized structures responsible for cannabinoid and terpene biosynthesis, making their density a critical parameter for both research and industrial applications. However, consistent trichome density assessment remains challenging due to anatomical variability and the absence of standardized methodologies. This review critically examines the existing literature on trichome quantification across key floral structures—such as bracts, sugar leaves, calyxes, and the main cola—to identify the most reliable sites and practices for accurate evaluation. Evidence suggests that bracts represent the most consistent sampling unit, given their homogeneous trichome distribution and elevated cannabinoid concentration. Whilst sugar leaves and calyxes are also frequently analyzed, their morphological variability requires cautious interpretation. Furthermore, trichome shape, size, maturity, and vegetal surface expansion/shrinkage during stress must be considered when correlating density with secondary metabolite production. We also highlight the advantages of using more than only one floral structure and integrating microscopic imaging and software-assisted analysis to enhance reproducibility and accuracy. By synthesizing current methodologies and proposing pathways for standardization, this review aims to support more robust trichome assessment protocols, ultimately improving cannabinoid yield optimization, quality control, broader cannabis research frameworks, and an important aesthetic parameter for consumers. Future research efforts should focus on advancing imaging methodologies and optimizing sampling protocols to further improve the precision and reproducibility of trichome density and cannabinoid analyses.

## 1. Introduction

### 1.1. Plant Trichomes

Virtually all terrestrial plant species possess epidermal outgrowths that resemble hair-like projections. These structures, commonly referred to as trichomes when located on aerial parts, are multifunctional adaptations that play a significant role in plant survival and adaptation [1]. When similar outgrowths occur on roots, they are typically referred to as root hairs, emphasizing their distinct physiological context. Etymologically, the term “trichomes” is derived from the Greek word “*trichos*” meaning hair, highlighting their morphological resemblance. Despite their superficial resemblance to vascular or ground tissues, trichomes are in fact specialized epidermal extensions rather than integral components of the plant’s internal vascular system [1].

Trichomes exhibit considerable diversity in form and function; they may be non-glandular, acting as physical barriers against herbivores and reducing water loss, or glandular, synthesizing and storing bioactive secondary metabolites such as essential oils, alkaloids, and terpenes [2]. Non-glandular trichomes can deter herbivores by creating physical barriers or by reducing palatability, whilst also regulating leaf temperature and water loss through light reflection—an adaptation particularly important in arid environments [3,4]. Meanwhile, glandular trichomes can synthesize a wide range of secondary metabolites, including essential oils, alkaloids, and terpenes, which serve defensive, allelopathic, or communicative functions [3]. Consequently, trichomes serve as a protective barrier against biotic threats (e.g., herbivores, pests, and pathogens) and abiotic stressors, including UV radiation and excessive transpiration. Interestingly, in particular species, they also facilitate seed dispersal or seed protection [4,5].

Structurally, trichomes are typically classified according to their cellularity (single-celled vs. multicellular), branching pattern (branched vs. unbranched), and secretory activity (glandular vs. non-glandular) [2]. They also take on diverse shapes such as star-like (stellate), hooked, peltate (scale-like), or capitate (headed). Larger, more conspicuous trichomes are often found on leaf undersides (abaxial surfaces) or along leaf edges, while smaller trichomes can appear in stomatal regions or in association with vascular bundles [6]. Notably, the presence of dense trichomes in some species increases epidermal thickness and may enhance the production of long-chain fatty acids, thus reducing water loss and stabilizing leaf temperature [7]. Recent transcriptomic studies suggest that the formation and specialization of trichomes are controlled by intricate genetic networks—highlighting a balance between the plant’s developmental signaling and environmental cues [5,8].

### 1.2. Trichomes in Cannabis sativa L.

**Figure 1:**
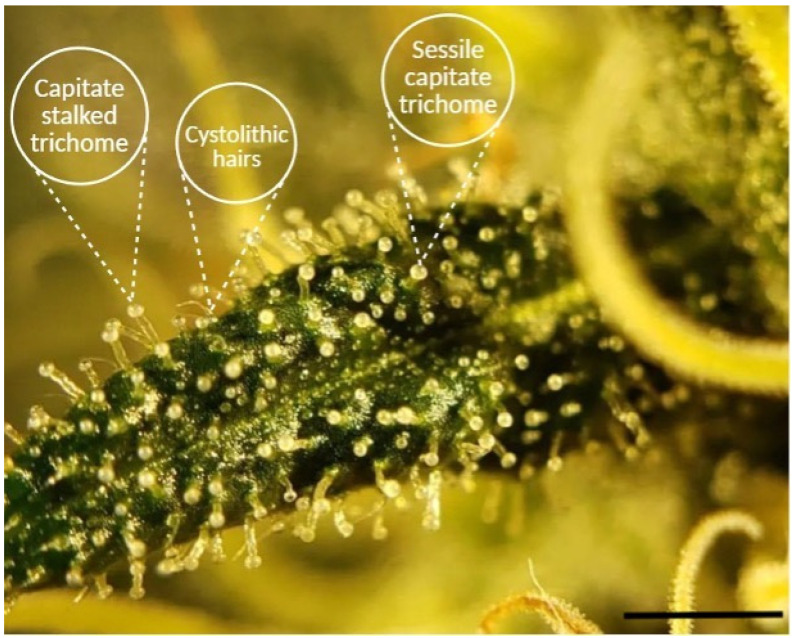
Zoomed image of a fresh *Cannabis sativa* sugar leaf surface showing capitate-stalked, sessile capitate, and cystolithic hairs, each indicated by labels and arrows created using BioRender (https://BioRender.com, accessed 10 April 2025). Original image courtesy of the authors. The scale bar represents 5 mm.

Among the numerous plant species that exhibit trichomes, *Cannabis sativa* L. stands out due to the medicinal and commercial significance of the metabolites produced in its glandular trichomes (Figure 1). Cannabis flowers, particularly those of female plants, have garnered attention due to their production of cannabinoids (e.g., Δ9-tetrahydrocannabinol [THC], cannabidiol [CBD]) and terpenes, compounds highly valued in both therapeutic and recreational contexts. The trichome biosynthesis of these specialized metabolites is influenced by the interplay of genetic factors, developmental stage, and environmental conditions, including light spectrum and intensity, temperature, and physical stress [9,10].

These compounds are synthesized and stored in glandular trichomes, which differ in structure, location, and metabolic function. On a morphological level, female cannabis flowers possess at least three distinct glandular trichome types based on their surface features and stalk length: bulbous, sessile capitate, and stalked (pedicellate) capitate [11].

- Bulbous trichomes are the smallest and are dispersed across various aerial surfaces of the plant. Despite their ubiquity, they remain the least characterized, partly due to their diminutive size.
- Sessile capitate trichomes have a more prominent glandular head and are typically abundant on sugar leaves and bracts.
- Stalked (pedicellate) capitate trichomes are generally larger and often regarded as the principal source of cannabinoids and terpenes within female inflorescences. These structures are most visible to the naked eye during the later flowering stages, creating the characteristic “frosty” appearance on colas and sugar leaves [10].

Although non-glandular structures such as cystolithic hairs are also present on cannabis leaf surfaces and included in Figure 1 for morphological reference, they are not involved in cannabinoid or terpene biosynthesis and therefore fall outside the focus of this review.

#### Morphological Distribution and Agronomic Relevance

Notably, bracts in female cannabis flowers frequently harbor higher densities of glandular trichomes and thus represent crucial sites for cannabinoid and terpene biosynthesis [10,12]. As a result, the distribution, density, and maturity of trichomes in specific floral regions—ranging from bracts and calyxes to sugar leaves—have become a focal point of breeding and cultivation strategies aimed at optimizing resin yield and the biochemical composition in terms of the bioactive compounds of interest. Beyond their biochemical significance, these trichomes offer protective functions, forming a defensive barrier against pests and environmental stresses during critical reproductive phases. Furthermore, higher trichome density creates a desirable aesthetic attribute for consumers.

Considering this dual role—both functional defense and bioactive compound biosynthesis and storage—trichomes in *C. sativa* L. are of paramount interest to researchers and cultivators. Understanding the morphological, genetic, biochemical, and environmental factors that govern trichome development and metabolism enables more precise cultivation practices and efficient breeding programs, ultimately influencing the quality and consistency of cannabis-derived products. The following sections delve deeper into the methods for assessing trichome density in various cannabis structures, intending to establish reproducible and standardized approaches in both research and industrial applications.

Trichome density is often considered an indicator of flower quality, particularly in relation to resin production and extraction yield. However, the lack of standardized protocols for measuring trichome density hinders comparison across studies and limits its reliability as a quality criterion in breeding and industry practices. Additionally, trichome distribution and morphology vary among floral structures—such as bracts, sugar leaves, calyces, and the main cola (inflorescence) —raising the question of which plant region best represents overall trichome density in analytical assessments.

### 1.3. Relevance of Trichome Density Assessment

The density and morphology of glandular trichomes vary markedly among different floral structures of *C. sativa*, including bracts, sugar leaves, calyxes, and the main cola. At the anatomical level, glandular trichomes house specialized secretory cells in their head region, which synthesize and store secondary metabolites such as cannabinoids and terpenes within a subcuticular cavity [5,11]. The accumulation of these metabolites is a dynamic process influenced by a confluence of factors—environmental conditions, developmental stage, and genetic background.

Recent genotype × environment (G × E) studies have shown that variations in light spectrum, temperature, humidity, and even UV-B light exposure can significantly modulate trichome density and secondary metabolite profiles [8,9]. Meanwhile, the age of the plant and the onset of the flowering stage can shift the trichome’s developmental trajectory, impacting both the density and chemical composition over time [10]. For instance, Punja et al. (2023) [10] noted that the number of capitate trichomes and the length of the pedicel differed significantly across genotypes and between the upper and lower surfaces of the bracts, underscoring the intricate interplay between genetics and plant morphology. These findings illustrate how structural variations in trichomes can affect the quality and consistency of the final product by influencing the content of cannabinoids and terpenes. Although the legacy cultivation community and empirical observations consider trichome density a reliable metric for cannabinoid levels, these findings suggest that trichome density alone is unlikely to be directly correlated with cannabinoid content, as trichome shape, size, maturity, and vegetal surface expansion/shrinkage during stress also play a significant role in this correlation. This review aims to avoid widespread biases and focus on validated scientific aspects in the literature.

**Figure 2:**
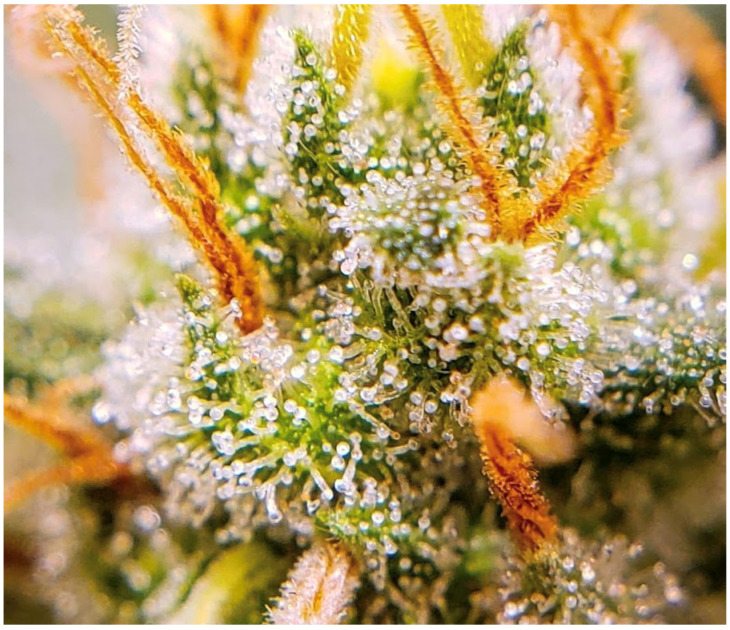
Details of the dense covering glandular trichomes that impart a “frosty” appearance to *Cannabis sativa* L. female flowers.

Beyond their biochemical relevance, trichomes also play a pivotal role in the visual and commercial appeal of *C. sativa* flowers, particularly in the recreational market. A dense covering of glandular trichomes imparts a characteristic “frosty” appearance to flowers, as shown in Figure 2, which is often perceived by consumers as an indicator of higher potency and quality. Consequently, breeders and cultivators target enhanced trichome coverage through selective breeding, aiming to maximize both resin yield and market value [11]. For medicinal applications, consistent trichome density across harvests helps ensure reproducible therapeutic efficacy. For recreational consumers, the visual allure can significantly influence purchasing decisions. Therefore, accurate assessment of trichome density is essential not only for research but also for quality control in commercial cultivation and product development.

### 1.4. Current Challenges and Knowledge Gaps

Despite its critical importance, accurately assessing trichome density remains a challenging task. Differences in trichome distribution and morphology across bracts, sugar leaves, calyxes, and main cola complicate comparisons, and the lack of standardized sampling and measurement protocols further limits reproducibility and inter-study consistency [5]. Additionally, the shape and size of trichomes influence metabolite content, indicating that simple counts alone may not fully capture their functional significance. A robust and reproducible methodology for trichome counting would therefore be of immense value, facilitating consistent assessments of cannabinoid yield and quality control in both research and industrial contexts. While the necessary imaging and computational technologies exist, their application for trichome quantification remains limited by a lack of focused research, standardized protocols, and validation efforts [8].

### 1.5. Review Objectives

This review aims to critically evaluate current methodologies for trichome density assessment in *Cannabis sativa*, with a focus on identifying the most reliable plant structures for sampling, evaluating quantification techniques, and highlighting the need for standardized protocols. By integrating morphological characterization with chemical profiling, this review aims to support advancements in cannabis research methodologies and industrial applications.

## 2. Morphological Targets for Trichome Quantification in Cannabis sativa

**Figure 3:**
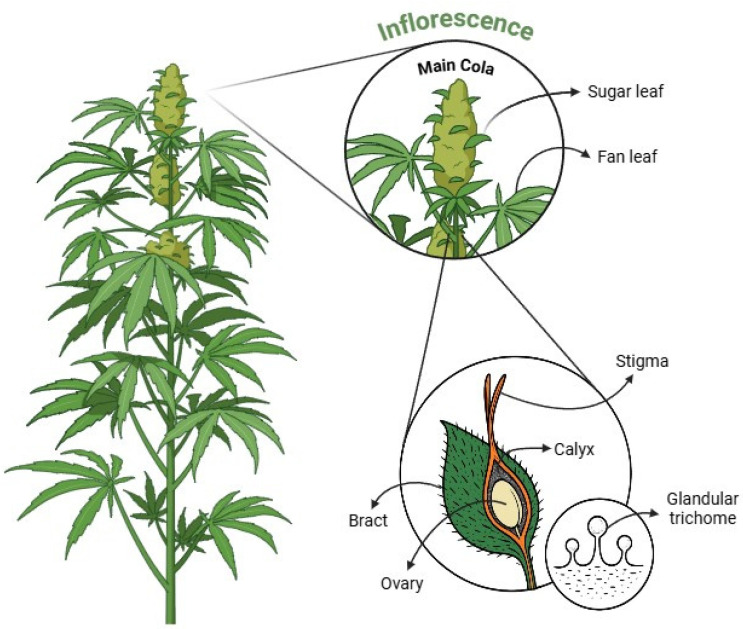
Illustration of cannabis’ anatomy highlighting anatomical regions for trichome identification, including main cola, sugar leaves, fan leaves, bracts, stigma, ovary, calyx, and trichomes. The figure was created using BioRender (https://BioRender.com, accessed 12 April 2025).

Accurate trichome quantification in *C. sativa* is often challenging due to the uneven distribution of trichomes across different plant structures. Consequently, researchers and cultivators use various methods to determine where and how to count trichomes. Regardless of the chosen method, consistent sampling protocols and clearly defined reference areas are vital for reproducibility. In the following sections, we examine how trichome density varies across key cannabis structures, as depicted in Figure 3, including the main cola, bracts, calyxes, and sugar leaves, and discuss the merits and limitations of focusing on each site for reliable trichome analysis.

### 2.1. Main Cola and Its Substructures for Trichome Analysis

**Figure 4:**
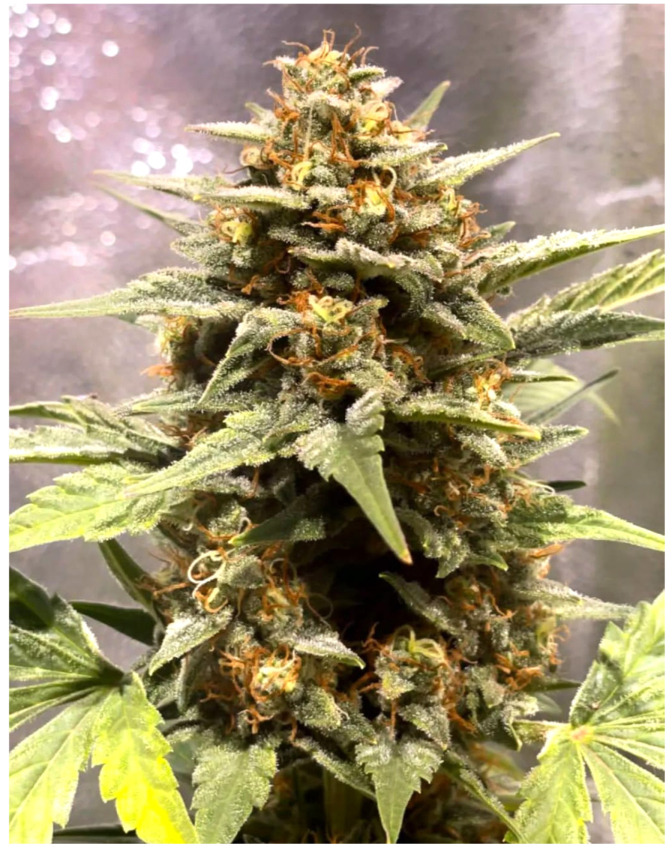
Representative image of the main cola of *Cannabis sativa* L., illustrating the organization of the inflorescence, the morphology of sugar leaves, and sections of fan leaves extending from the floral cluster.

As illustrated in Figure 4, the main cola, or apical bud, stands out as the plant’s primary flowering site and is frequently cited as having one of the highest trichome densities [13]. This dense coverage is often attributed to favorable light exposure at the canopy’s apex, and with preferential nutrient allocation during flowering. However, trichome distribution within the main cola is not uniform, reflecting micro-environmental variations (e.g., light penetration, airflow) and internal plant dynamics [9]. To address this variability, researchers commonly recommend standardized sampling protocols, such as selecting a consistent 1–2 cm section of the upper cola [10]. This approach helps capture a comparable physiological zone across samples, reducing intra- and inter-plant variability.

Importantly, even when focusing on the main cola, it is necessary to define which specific substructures—such as upper bracts, calyxes, or sugar leaf tips—are being analyzed. Providing the definition enhances accuracy in trichome quantification and facilitates cross-study comparisons. By combining well-defined sampling sites with digital imaging tools, researchers can further reduce observer bias and obtain more reliable data.

### 2.2. Bracts

**Figure 5:**
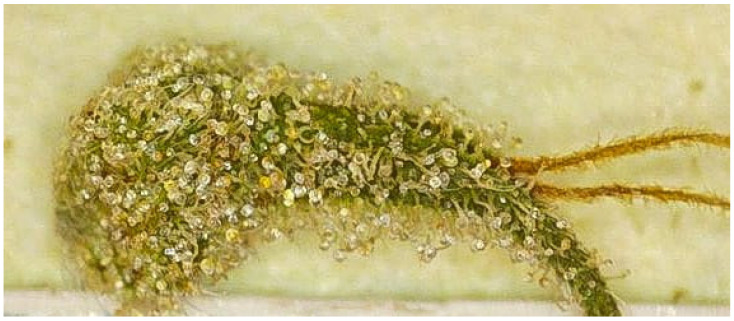
High-magnification microscopic image of glandular stalked capitate trichomes on the surface of bracts from a dried female *Cannabis sativa* L. inflorescence.

Bracts in cannabis are small, leaf-like structures that surround and protect the reproductive organs of the plant (Figure 5). They are often described as the most reliable site for trichome analysis due to their dense and homogeneous resin coverage. Unlike other floral structures, bracts display a relatively consistent distribution of glandular trichomes, making them particularly suitable for microscopic imaging and cannabinoid quantification [12]. In addition, bracts are larger and more exposed than inner floral parts; they provide an accessible and efficient target for cannabinoid research. This accessibility streamlines the quantification of cannabinoids and terpenes, which in turn facilitates both breeding research and commercial quality control efforts [5].

The protective role of bracts aligns with their high density of glandular trichomes. The secondary metabolites produced in these trichomes not only deter herbivores and pathogens but also form the basis for the plant’s medicinal and recreational value [10]. As a result, focusing on bracts offers researchers and cultivators insight into one of the primary sites of cannabinoid and terpene biosynthesis. This dual role makes bracts a particularly appealing target for those aiming to optimize resin output or investigate the genetic and physiological mechanisms of trichome development.

By combining consistent bract sampling with rigorous imaging protocols, studies can achieve high reproducibility of trichome density measurements, an essential requirement for both breeding programs and product standardization. For these reasons, bracts represent one of the most reliable benchmarks for comparing genotypic variations and environmental influences on trichome production in *C. sativa*.

### 2.3. Calyxes

Although the term *bract* is sometimes used interchangeably with *bractlet* or *calyx*, botanical classifications distinguish bracts as distinct structures that envelop the developing flowers and seeds [14]. Calyx’s primary function is to protect the plant’s reproductive components, and it generally lacks the glandular trichomes abundant on the surrounding bracts. The term *calyx* in *C. sativa* remains a point of contention, as some sources equate it with the bract. In contrast, others view it as a distinct inner structure that encases the ovule [14,15], reflecting overlapping morphology and inconsistent usage in the literature. Nevertheless, the generally accepted definition positions the calyx as an inward, protective layer situated within the bracts, playing a critical role in the development of the reproductive organs.

Despite logistical challenges, calyxes are important due to their role in enclosing the ovules. However, since calyxes potentially lack the glandular trichomes responsible for resin production, their direct contribution to cannabinoid and terpene biosynthesis is limited compared to bracts. Therefore, any methodologies referring to trichome analysis on calyxes likely involve bracts due to the morphological confusion and the absence of glandular trichomes on the true calyx. It is also important to note that, to date, there are no standardized studies on sampling and quantification protocols specifically for calyxes, distinct from those used for bracts, which contributes to variability in reported data.

### 2.4. Sugar Leaves

**Figure 6:**
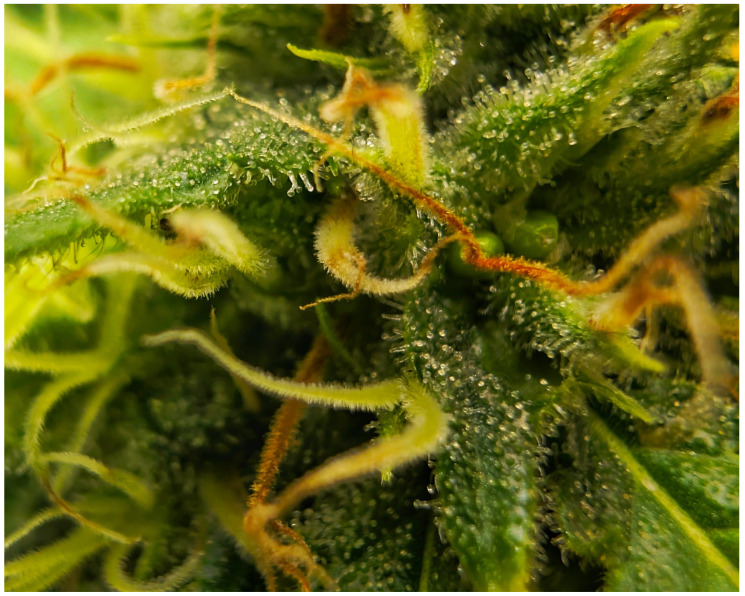
High-magnification image of immature glandular trichomes on the surface of sugar leaves from a fresh *Cannabis sativa* L. inflorescence, illustrating the early stages of resinous biomass production.

Sugar leaves, as shown in Figure 6, are the small, “frosty” leaves that surround cannabis buds and represent another key site for trichome density analysis. These leaves can contribute significantly to overall trichome yield [15], with higher densities observed closer to their tips, an attribute that makes them particularly valuable for cannabinoid extraction. In addition to their role as trichome-bearing structures, sugar leaves retain some photosynthetic capacity, which may support local energy and nutrient supply for developing buds and resin production [11]. Although their photosynthetic contribution is modest compared to larger fan leaves, the combination of limited photosynthesis and high trichome content in sugar leaves makes them an intriguing focus for both morphological and biochemical analyses in cannabis research.

Sugar leaves exhibit a less uniform distribution of trichomes than bracts, with elevated densities being found at the margins and tips. This variability requires meticulous sample techniques, often focusing on areas rich in trichomes, to ensure precise density assessments. Whilst sugar leaves lack the uniformity of bracts, their inclusion in trichome analysis offers valuable supplementary data, especially for evaluating whole-plant cannabinoid concentration and understanding spatial variability in trichome production across different leaf areas and developmental stages.

## 3. Standardization and Sampling Considerations for Reliable Trichome Quantification

### 3.1. Manual Methods

Manual analyses of trichomes often involve imaging the surface under a dissecting or compound microscope, followed by manual counting of individual glandular structures [5]. Whilst widely used, this approach is labor-intensive and susceptible to observer bias [10]. These limitations can reduce reproducibility across studies, particularly when sampling areas are inconsistently defined.

### 3.2. Semi-Automated Methods

To address this, researchers increasingly employ digital imaging tools, such as ImageJ-1.54p software (ImageJ, U.S. National Institutes of Health, Bethesda, MD, USA, https://imagej.net/ij/), which allow for standardized area selection (e.g., 1 mm^2^) and semi-automated trichome quantification. ImageJ and similar platforms help reduce user bias while improving reproducibility in trichome counts across experimental replicates [5].

### 3.3. Advanced Laboratory Methods

Confocal and scanning electron microscopy (SEM) provide high-resolution visualization of trichome morphology and distribution [5]. However, these techniques are rarely applied in quantitative cannabis studies, and there is a lack of validated protocols or large-scale studies employing these tools for standardized trichome density assessments. Also, the cost of these pieces of equipment is a limiting factor; as such, their use remains largely illustrative.

### 3.4. Field-Based Approaches

Field-based methods, such as hand lenses, macro-lens photography, or portable microscopes, are frequently used by cultivators for qualitative assessments of trichome maturity—for example, observing color changes (clear, cloudy, amber) to inform harvest timing. These tools provide fast, non-invasive visual cues; however, they are not validated for quantitative trichome density analysis and are not suitable replacements for laboratory methods when accurate quantification is required.

## 4. Discussion

Establishing reliable methods for trichome density analysis remains central to *Cannabis sativa* L. research, given that secondary metabolites, primarily cannabinoids and terpenes, accumulate in these glandular structures.

Whilst the present review focuses on identifying reliable sampling targets for trichome quantification, it also highlights the methodological gaps that hinder standardization. Current approaches are predominantly manual or semi-automated, relying on dissecting microscopy and software for counting trichomes in defined areas. Although advanced tools, such as scanning electron microscopy, confocal imaging, and machine learning-based detection, offer high-resolution and high-throughput alternatives, they remain underutilized and largely unvalidated in cannabis research. Comparative studies employing multiple quantification techniques across cultivars and growth conditions are still lacking, limiting confidence in cross-study reproducibility. Therefore, despite progress in imaging and computational tools, the field urgently requires consensus on validated, reproducible methods tailored to cannabis trichome analysis.

Among the plant tissues evaluated, bracts consistently demonstrate the highest reproducibility due to their relatively uniform trichome distribution and significant cannabinoid content [10]. This consistency also translates into lower plant-to-plant variance, a critical attribute for industrial-scale applications, where maintaining batch consistency directly impacts market value and therapeutic efficacy [11].

Still, sugar leaves and calyxes offer valuable perspectives depending on experimental objectives. Sugar leaves, although less uniform in trichome dispersion, can illuminate extraction efficiency since their edges and tips often host abundant glandular heads [15]. Calyxes, despite posing sampling challenges due to their smaller size and proximity to reproductive tissues, represent strategic focal points for investigating cannabinoid biosynthesis pathways [14]. In contrast, although visually prominent, the main cola can exhibit substantial variability in trichome coverage; hence, standardization for this region typically involves precisely defined substructures (e.g., uppermost bracts) rather than the entire apical bud [13].

Despite trichomes’ pivotal role, higher density alone does not guarantee greater cannabinoid potency [12]. Recent studies underscore that while trichome density is a valuable morphological marker, it does not consistently predict cannabinoid content across genotypes. Multiple studies, including recent metabolomic and transcriptomic work, confirm that biosynthetic enzyme activity, genotypic expression profiles, and resin storage capacity are equally, if not more, influential in determining the final amounts of cannabinoids [8,10]. A cultivar with moderate trichome density but upregulated synthase genes may surpass a denser but genetically less active cultivar in total cannabinoid contents. Consequently, relying solely on trichome counting could obscure the true biochemical potential of a given plant genotype. Integrating morphological data (trichome density, distribution, shape, and size) with chemical assays (cannabinoid and terpene identification and quantification) thus provides a more robust assessment of cannabis potency and value.

From a breeding standpoint, uniform and precise trichome density metrics enable phenotypic selection for cultivars that exhibit stable, high-resin traits under diverse environmental conditions [9]. Furthermore, environmental factors such as plant density and architecture significantly influence cannabinoid uniformity. Studies indicate that higher planting densities can reduce cannabinoid uniformity across different parts of the plant, although they may increase overall yield per area. Architectural modifications, such as defoliation or pruning, have been shown to mitigate some of these effects, thereby enhancing chemical standardization [16].

Moreover, anatomical and morphological differences among cannabis trichomes have been linked to variations in metabolite profiles. Microcapillary sampling of individual glandular trichomes revealed that stalked trichomes on floral tissues predominantly accumulate monoterpenes, with monoterpene-to-sesquiterpene ratios exceeding 12:1. In contrast, sessile trichomes on vegetative leaves exhibited lower monoterpene content and higher sesquiterpene proportions. Interestingly, despite these differences in terpene profiles, cannabinoid compositions between stalked and sessile trichomes were found to be similar. These findings underscore the importance of considering trichome type and sampling location when analyzing secondary metabolite profiles, as morphological variations can significantly influence terpene composition, whilst cannabinoid content may remain relatively consistent across trichome types. The study by Livingston et al. (2020) [11] examines the dynamic changes in cannabis trichome morphology and their relationship with metabolite content throughout the plant’s maturation, offering insights into the underlying mechanisms that drive metabolite synthesis.

Adding to this, Ferri et al. (2024) [17] demonstrated that even individual glandular trichomes isolated from the same cannabis flower can display detectable variations in their phytocannabinoid profiles. In their study, single trichomes from two *Cannabis sativa* L. varieties—a medicinal strain (FM2) and a CBD-rich hemp cultivar—were analyzed using an untargeted metabolomics approach. Around 70 phytocannabinoids, including carboxylated and decarboxylated forms of CBD, THC, and CBG, were identified. Although the profiles of isolated trichomes and whole inflorescences were broadly similar, absolute cannabinoid levels varied among trichomes, while the CBD-to-THC ratio remained consistent, reflecting genetic chemotypes. These results highlight that chemical variability exists even within a single anatomical region, suggesting that differences across broader plant structures, such as leaves, bracts, and flowers, could be even more significant. Despite the overall consistency observed between isolated trichomes and whole flower extracts, subtle differences reinforce the hypothesis that cannabinoid biosynthesis is influenced not only by genetics but also by tissue-specific factors. Therefore, taking into account more than one floral structure mentioned in this review could increase trichome density reliability when correlating it with total cannabinoid concentration.

Despite ongoing advancements in microscopic imaging and software-assisted quantification, standardizing sampling protocols still faces significant hurdles. Variability introduced by genotype, developmental stage, and environmental factors often leads to inconsistent data across research labs and commercial operations [5,9]. Moreover, cultivars can manifest unique developmental timelines for trichome initiation, and environmental stressors (e.g., UV-B light, nutrient shifts) can differentially modulate secondary metabolite biosynthesis and accumulation [8]. Such variability underscores the need for harmonized guidelines, including standardized sampling areas (e.g., 1 mm^2^ or 2 mm^2^ sectors), consistent plant growth stages (e.g., late flowering or post-harvest), and documented environmental parameters (e.g., light intensity and humidity ranges).

Microscopic imaging (optical, confocal, electron) combined with macro-photographic methods improves the resolution and accuracy of trichome assessments. Platforms like ImageJ or custom machine learning algorithms help reduce observer bias, permitting faster and more reproducible data collection [5]. However, uniform calibration of imaging equipment and consistent magnification levels are crucial for cross-study comparability [10]. Tanney et al. (2021) [5] demonstrated that portable Raman spectroscopy can non-destructively and rapidly profile cannabinoids and terpenes in trichomes without extensive sample preparation, offering a powerful tool for real-time, in situ analysis. In parallel, machine learning approaches have been utilized to automate trichome identification and quantification from microscopic images, as demonstrated by Ebersbach et al. (2018) [18] in Arabidopsis, thereby improving throughput and reducing observer bias—methods that could be adapted for cannabis research. Comparative studies employing multiple quantification techniques—such as manual counting, ImageJ analysis, and AI-based methods—across various cannabis cultivars under controlled conditions are warranted to validate the reliability and reproducibility of these approaches.

As regulatory frameworks evolve worldwide, robust methods for trichome evaluation will be increasingly important not only for academic research but also for quality control in commercial settings. Validated trichome density assays may become integral to legal compliance, product labeling, and ensuring batch-to-batch consistency, particularly in medicinal markets where patients rely on reproducible dosage [11]. Also, it creates a metric for a trait that is desirable to consumers. The development and adoption of standardized best practices will be essential for advancing *Cannabis sativa* as a scientifically validated crop.

## 5. Conclusions

Establishing standardized methods for trichome density assessment remains a critical priority for advancing cannabis research.

This review highlights that, among different plant tissues, bracts stand out as the most reliable sampling site due to their consistent glandular coverage and high cannabinoid concentrations—key attributes for reproducible and robust quantification. Nevertheless, morphological evaluations alone are insufficient to fully capture the potency and chemical variability of cannabis. Moreover, using several floral structures or tissues could increase the relevance and accuracy of the whole-plant cannabinoid total concentration. Future methodologies must integrate trichome density, distribution, shape, and size metrics with biochemical profiling, thereby reflecting not only anatomical structure but also tissue-specific metabolic potential. Advanced imaging technologies, automated quantification tools, and harmonized sampling protocols will be crucial for enhancing reproducibility across both research and industrial applications. As the cannabis industry matures and regulatory frameworks evolve, validated trichome analysis methods will play an increasingly central role in quality control, cultivar selection, and therapeutic product development. Building a foundation of validated, multidimensional assessment practices, including morphological, chemical, and computational tools, will be key to unlocking the full scientific and commercial potential of *Cannabis sativa*.

## Data Availability

The original contributions presented in the study are included in the article; further inquiries can be directed to the corresponding authors.
